# Coordination between aminoacylation and editing to protect against proteotoxicity

**DOI:** 10.1093/nar/gkad778

**Published:** 2023-09-23

**Authors:** Hong Zhang, Parker Murphy, Jason Yu, Sukyeong Lee, Francis T F Tsai, Ambro van Hoof, Jiqiang Ling

**Affiliations:** Department of Cell Biology and Molecular Genetics, The University of Maryland, College Park, MD 20742, USA; Department of Cell Biology and Molecular Genetics, The University of Maryland, College Park, MD 20742, USA; Department of Cell Biology and Molecular Genetics, The University of Maryland, College Park, MD 20742, USA; Department of Biochemistry and Molecular Biology, Baylor College of Medicine, Houston, TX 77030, USA; Advanced Technology Core for Macromolecular X-ray Crystallography, Baylor College of Medicine, Houston, TX 77030, USA; Department of Biochemistry and Molecular Biology, Baylor College of Medicine, Houston, TX 77030, USA; Advanced Technology Core for Macromolecular X-ray Crystallography, Baylor College of Medicine, Houston, TX 77030, USA; Department of Molecular and Cellular Biology, Baylor College of Medicine, Houston, TX 77030, USA; Department of Molecular Virology and Microbiology, Baylor College of Medicine, Houston, TX 77030, USA; Department of Microbiology and Molecular Genetics, The University of Texas Health Science Center at Houston, Houston, TX 77030, USA; Department of Cell Biology and Molecular Genetics, The University of Maryland, College Park, MD 20742, USA

## Abstract

Aminoacyl-tRNA synthetases (aaRSs) are essential enzymes that ligate amino acids to tRNAs, and often require editing to ensure accurate protein synthesis. Recessive mutations in aaRSs cause various neurological disorders in humans, yet the underlying mechanism remains poorly understood. Pathogenic aaRS mutations frequently cause protein destabilization and aminoacylation deficiency. In this study, we report that combined aminoacylation and editing defects cause severe proteotoxicity. We show that the *ths1-C268A* mutation in yeast threonyl-tRNA synthetase (ThrRS) abolishes editing and causes heat sensitivity. Surprisingly, experimental evolution of the mutant results in intragenic mutations that restore heat resistance but not editing. *ths1-C268A* destabilizes ThrRS and decreases overall Thr-tRNA^Thr^ synthesis, while the suppressor mutations in the evolved strains improve aminoacylation. We further show that deficiency in either ThrRS aminoacylation or editing is insufficient to cause heat sensitivity, and that *ths1-C268A* impairs ribosome-associated quality control. Our results suggest that aminoacylation deficiency predisposes cells to proteotoxic stress.

## Graphical Abstract

**Graphical Abstract:**
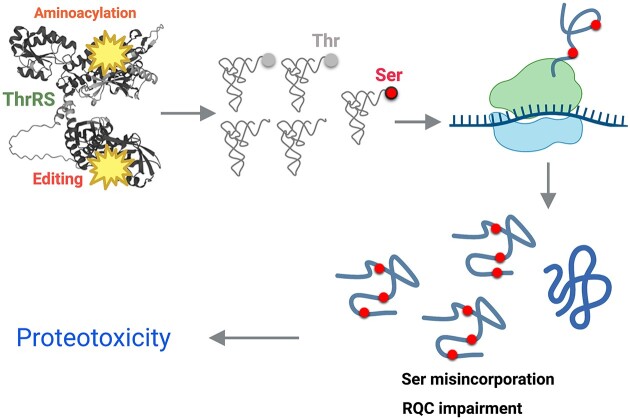

## INTRODUCTION

The genetic code is established through accurate pairing between aminoacyl-tRNAs (aa-tRNAs) and mRNA codons on the ribosome (1–4). Except for selenocysteine, each of the 22 natural proteinogenic amino acids is recognized by a specialized aminoacyl-tRNA synthetase (aaRS) and ligated to the corresponding tRNAs through an aminoacylation reaction (5,6). Efficient aminoacylation is thus critical to supply the ribosome with aa-tRNAs for translation. In addition to aminoacylation, many aaRSs also utilize an editing step to prevent the mispairing of amino acids and tRNAs that would otherwise result in protein mistranslation and misfolding (7,8). Editing can occur before or after amino acid transfer to the tRNA by the aaRS in *cis* or by a separate *trans*-editing factor (9).

Given the essential role of aaRSs in protein synthesis, it is unsurprising that mutations in aaRS genes are widely associated with human diseases. Autosomal dominant mutations in six aaRSs have been shown to cause a peripheral neurodegenerative disorder known as Charcot-Marie-Tooth (CMT) disease (10–13). CMT mutations in glycyl- (GlyRS) and alanyl- (AlaRS) tRNA synthetases do not affect aminoacylation but induce aberrant interactions with Nrp1 to impair signal transduction in neurons (13,14). Recent studies in a mouse model suggest that CMT mutations in GlyRS also activate the integrated stress response (ISR) to attenuate protein synthesis and cause neural toxicity (15,16). Inhibiting ISR or overexpressing tRNAs alleviates phenotypes associated with certain CMT mutations (16–18). On the other hand, biallelic recessive mutations have been identified in most cytoplasmic aaRSs to cause disorders in the central nervous system, such as microcephaly and seizure (11,12,19–21). These homozygous or compound heterozygous mutations frequently lead to aminoacylation defects or protein instability.(11) How these apparent loss-of-function mutations cause defects at the cellular level remains poorly understood.

AlaRS and threonyl-tRNA synthetase (ThrRS) belong to the Class II aaRSs and share a similar editing site to hydrolyze misacylated Ser-tRNA^Ala^ and Ser-tRNA^Thr^, respectively (22–27). We previously showed that compound heterozygous mutations in AlaRS impair aminoacylation and editing and cause microcephaly with severe neuron degeneration in children (21). Additionally, AlaRS editing-defective mutations in mice cause neurodegeneration and cardioproteinopathy (28,29). In human patients, ThrRS editing-site mutations are associated with intellectual disability and lead to destabilization of the ThrRS protein (30). Despite such evidence from human patients and animal models, whether editing defects directly contribute to human diseases remains unknown.

In addition to being used by the ribosome during canonical protein synthesis, Thr-tRNA^Thr^ and Ala-tRNA^Ala^ synthesized by ThrRS and AlaRS are also used in the ribosome-associated quality control (RQC) pathway (31–33). RQC senses stalled ribosomes and adds C-terminal Ala and Thr (CAT) tails to stalled polypeptides. Formation of the CAT tail does not require an mRNA template and facilitates degradation or sequestration of truncated polypeptides, thus protecting cells against proteotoxicity (31,34–37). Impairing CAT tailing results in neurological defects in mice and humans (38).

In this study, we wished to understand the cellular impact of ThrRS editing deficiency in eukaryotes. We used genome editing to introduce the *ths1-C268A* mutation to abolish editing of cytoplasmic ThrRS in *Saccharomyces cerevisiae*. The equivalent mutation has been shown to be essential for ThrRS editing in bacteria (24,39). The *ths1-C268A* mutation in yeast led to Ser misincorporation at Thr codons and resulted in a severe growth defect under heat stress. We next evolved suppressor mutations that restored heat resistance in the *ths1-C268A* strain. Unexpectedly, all suppressor mutations mapped to the *THS1* gene that encodes the cytoplasmic ThrRS. Further *in vitro* biochemical analyses revealed that the suppressor mutations increased the aminoacylation efficiency but did not rescue the editing defect of ThrRS. Similar to some aaRS mutations in human patients, we show that the *ths1-C268A* mutation destabilizes ThrRS and leads to a lower tRNA^Thr^ aminoacylation level, which in turn impairs both global protein synthesis and the RQC pathway. We further show that decreasing ThrRS aminoacylation or editing efficacy alone is insufficient to cause proteotoxicity. Our work thus uncovers previously unknown coordination between aminoacylation and editing to protect cells against proteotoxic stress.

## MATERIALS AND METHODS

### Plasmids and strains

All *Saccharomyces cerevisiae* strains used here were derivatives of BY4741. *Escherichia coli* DH5α grown in Luria Broth (LB) medium was used for molecular cloning. Yeast cells were grown in YPD medium (1% yeast extract, 2% peptone, and 2% glucose) or Synthetic Defined (SD) dropout medium (0.17% yeast nitrogen base, 0.5% ammonium sulfate, 2% glucose, and 0.14% amino acid dropout mix, -His, -Leu or -Ura). Mutant yeast strains were obtained using CRISPR/Cas9 (40). Briefly, pCas9 was transformed into the WT strain and selected on SD-Leu plates. Next, psgRNA targeting the *THS1* gene and donor DNA fragments were electro-transformed into competent cells. All transformants were selected on SD-Leu-His agar plates. Colonies were verified by PCR and Sanger sequencing. To obtain the mutant library with varying expression levels of ThrRS, we used CRISPR/Cas9 to mutate the DNA sequence 5′ to the initiation codon of *THS1* from CAGAGCAAGAAAATAAAACGGAG to TAGGGCTAGGAAGTATAANNNNN (N represents A/T/C/G). To create *gcn2*Δ, *ltn1*Δ and *rqc2*Δ mutants, the coding regions were replaced with the *LEU2* or *HIS3* gene and verified by PCR. The strains and plasmids are listed in Table S2, and the primers used are listed in Table S3.

### Growth assays

Single colonies of yeast strains were grown in YPD at 30°C to saturation, and diluted at 1:50 in YPD in a 96-well plate. Continuous growth at 30 or 37°C was monitored with a microplate reader (BioTek Synergy H1). For the spotting assay, single colonies were grown to saturation in YPD at 30°C, diluted at 1:50 in YPD, and grown to log phase. Cell cultures were then serially diluted, spotted on YPD agar plates, and incubated both at 30°C and 37°C for 2–4 days before imaging.

### β-Lactamase assay

To determine Ser misincorporation at Thr codons, yeast cells carrying the β-lactamase gene *bla*, *bla-S68T* or the empty vector (pJD1212 or pRS316) were grown in SD-Ura to log phase and adjusted to the same *A*_600_. 1 ml of each culture was collected and washed with phosphate-buffered saline (PBS), and the pellets were resuspended in 200 μl lysis buffer (PBS pH 7.0, 1 mM phenylmethylsulfonyl fluoride (PMSF), 10 mM dithiothreitol (DTT), 1× protein inhibitor cocktail, and 3 mg/ml zymolyase) and incubated at room temperature for 40 min with vortexing. β-Lactamase activity was measured in a 100 μl reaction mixture containing PBS pH 7.0, 100 μM nitrocefin, and cell lysate (10 μl for the strains carrying *bla-S68T*, and 0.1 μl for the strains carrying WT *bla*). A486 absorption was recorded with a microplate reader (BioTek Synergy H1). The WT β-lactamase (with Ser at position 68) shows >10 000-fold higher activity than the S68T mutant in the WT yeast strain. To calculate the Ser misincorporation rate of each yeast strain, the β-lactamase activity of *bla-S68T* was divided by that of the WT *bla*.

### Experimental evolution of suppressor mutants

A scheme of experimental evolution is shown in Figure S3A. Briefly, three single colonies of the *ths1-C268A* strain were inoculated in separate tubes in YPD and grown for 2 days to saturation at 30°C. For each round of evolution, cell cultures were diluted at 1:100 in YPD and grown at 37°C for 2 days to saturation. A fraction of the culture after 10 and 15 rounds of evolution was streaked on YPD plates to isolate single colonies for growth analysis and sequencing.

### Genome sequencing

Genome sequencing was performed by Azenta Life Sciences and analyzed as described (41). PE150 libraries of the yeast strains were prepared, sequenced, and mapped by Azenta Life Sciences. Reads are deposited in Sequence Read Archive (SRA) under accession number PRJNA972286. To identify mutations, we used FreeBayes (https://arxiv.org/abs/1207.3907) for the detection of candidate variants. The Integrated Genome Viewer (https://software.broadinstitute. org/software/igv/download) was used to inspect candidate variants. True mutations were differentiated from sequencing errors and preexisting SNPs by being supported by the consensus of the reads in the evolved isolate(s), but not by the reads from the WT or starting *ths1-C268A* strain. Aneuploidy was detected by generating read coverage over a sliding 10K window with the Bamcoverage tool (42) and inspecting the resulting bigwig files in the IGV viewer.

### Aminoacylation and deacylation assays *in vitro*

His_6_-tagged yeast ThrRS variants were expressed and purified from *E. coli* Rosetta (DE3) pLysS. Aminoacylation assays were performed as described with slight modifications (39). Briefly, Thr aminoacylation activity was determined using 0.3 μM ThrRS, 5 mg/ml total yeast tRNAs containing ∼3 μM of tRNA^Thr^ (Roche), 4 mM ATP, and 18 μM ^14^C-Thr in aminoacylation buffer (100 mM Na-HEPES pH 7.2, 30 mM KCl and 10 mM MgCl_2_) at 37°C. At each time point, aliquots were spotted on 3MM Whatman paper discs pre-soaked with 5% trichloroacetic acid (TCA), washed three times with 5% TCA, dried, and their radioactivity level was measured with a scintillation counter. Ser misacylation activity was similarly determined, except that 3 μΜ ThrRS and 50 μM ^14^C-Ser were used instead.

For deacylation, first, ^14^C-Ser-tRNA^Thr^ was prepared. Briefly, 5 μM ThrRS C268S, 2 mM ATP, 50 μM ^14^C-Ser, 5mM DTT and 10 mg/ml total yeast tRNAs (Roche) were added in a 500 μl reaction containing 100 mM Na-HEPES pH 7.2, 30 mM KCl and 10 mM MgCl_2_. The reaction was stopped with the addition of 125 mM sodium acetate pH 4.5 after 10 min at 37°C, and extracted with acidic phenol/chloroform. After ethanol precipitation and wash, the tRNA pellet was dissolved in 50 μl RNase-free water. Deacylation was performed by incubating 50 nM of above-prepared ^14^C-Ser-tRNA^Thr^ with 1 μΜ ThrRS in 100 mM Na-HEPES pH 7.2, 30 mM KCl and 10 mM MgCl_2_ at 37°C.

### Western blot

A FLAG tag was inserted into the C-terminus of *THS1* using CRISPR/Cas9. Total proteins were extracted from overnight cultures and precipitated with TCA. The same A600 of cells were collected and suspended in 1 ml of ice-cold NaOH (0.3 M) and 1% β-mercaptoethanol, and then incubated on ice for 15 min with occasional vortexing. TCA was then added to a final concentration of 10%, and the samples were incubated on ice for 15 min with occasional vortexing. The mixtures were centrifuged at 13 000 rpm for 20 min, and the supernatants were carefully removed. The pellets were resuspended 100 μl loading buffer (8 M urea, 5% SDS, 200 mM Tris pH 6.8, 1 mM EDTA, and 100 mM DTT with bromophenol blue) and heated for 15 min before separation by 12% SDS-PAGE and western blot. The antibody dilutions used were: 1:2000 for the mouse monoclonal anti-FLAG antibody (Sigma-Aldrich), 1:5000 for the mouse monoclonal anti-PGK1 primary antibody (Invitrogen), and 1:5000 for the goat-anti-mouse IgG-HRP secondary antibody (Invitrogen). Nitrocellulose membranes were treated with enhanced chemiluminescent substrate reagents (Bio-Rad) and visualized using a ChemiDoc Imaging System (Bio-Rad).

### RQC analysis

Yeast cells carrying pTDH3-GFP-R12-RFP(34) were grown in SD-Ura. Cell pellets were resuspended in PBS and adjusted to the same *A*_600_. The total proteins were extracted as described above and separated on a 12% SDS-PAGE gel. A standard western blot protocol was applied as described above. The mouse anti-GFP first antibody (Invitrogen) was used with 1:1000 dilution.

### Acidic northern blot

Total RNAs from yeast cells were extracted using a hot-phenol method. Overnight cultures were treated at 37°C for 2 h, pelleted, and resuspended in 0.3 M sodium acetate pH 4.5. One volume of acidic phenol was then added. The samples were vortexed for 10 s and incubated on ice for 15 min with occasional vortexing. The aqueous phase was collected after centrifugation at 13 000 rpm for 15 min at 4°C. Two volumes of ice-cold 100% ethanol were added, and the samples were kept at −80°C for 1 h. Pellets were collected by centrifugation for 20 min at 13 000 rpm, washed with 70% ethanol, dried at room temperature for 5 min, and dissolved in sodium acetate buffer (pH 5.2). Total RNAs were separated on an acidic urea gel prepared with 0.1 M sodium acetate buffer pH 5.2. Deacylated tRNA^Thr^ control was obtained by treating the RNA samples in Tris buffer pH 9.0 for 1 h at 42°C. For northern blot, the nylon membrane was treated with Ultrahyb™ hybridization buffer (Invitrogen) at 42°C for 1 h after UV crosslinking, and incubated with 100 ng/μl biotin-labeled DNA probe (Biotin-TTGAACCGATGATCTCCACA) at 42°C overnight. Finally, tRNA^Thr^ and aa-tRNA^Thr^ were detected using the Chemiluminescent Nucleic Acid Detection Kit (Thermo Fisher Scientific).

### Overall protein synthesis assay

The protein synthesis assay was carried out as described (43) using a methionine analog (L-homopropargylglycine (HPG) containing an alkyne moiety. Yeast cells were grown to log-phase in YPD, adjusted to the same *A*_600_, washed twice with SD-Met medium, and incubated with 100 μl SD-Met for 1 h. Fixation and fluorophore labeling were performed according to the manufacturer's protocol (Click-iT® HPG Alexa Fluor® 594 protein synthesis assay kit, Thermo Fisher), and the fluorescence was determined using a Synergy HT microplate reader. The fluorescence signal indicates the overall synthesis activity of the proteome within the 1-h time frame. The fluorescence signals of the mutants were normalized with that of the WT (set at 1).

### Generating a 3D homology model for yeast cytoplasmic ThrRS with bound tRNA

The cytoplasmic ThrRS from *Saccharomyces cerevisiae* (P04801) is an 84.5-kDa protein comprising 734 amino acid residues, which forms a dimer that binds two molecules of tRNA (44). Because The 3D structure of *Sc*ThrRS is unknown, we used HHpred (45) to identify structural homologs of *Sc*ThrRS present in the PDB_mmCIF70_10_Jan structural database. The bioinformatic analysis yielded 169 structure hits reported in the protein data bank (www.rcsb.org), which mostly consisted of members of the aminoacyl-tRNA synthetase family. The top solution was found with *Escherichia coli* ThrRS (PDB: 1QF6; *E*-value: 1.7e-77; Score 697.52), whose crystal structure was determined to 2.9-Å resolution with bound tRNA (46) and which was used as input into MODELLER (47) without the tRNA coordinates.

The resulting 3D model of *Sc*ThrRS (residues 74–729) was inspected using COOT 0.8.9.2 (48) and superposed onto the crystal structure of *Ec*ThrRS (PDB: 1QF6_A), which revealed steric clashes between *Sc*ThrRS and the superposed tRNA. To remove short contacts, *Sc*ThrRS was split into three rigid bodies consisting of residues 74–328, 329–619 and 620–729. Each rigid body was superposed individually onto the crystal structure of the *Ec*ThrRS monomer. The superposed rigid bodies were visually inspected for steric clashes and then manually connected to generate a 3D model for the *Sc*ThrRS monomer. The *Sc*ThrRS dimer was generated by applying the same 2-fold crystallographic symmetry matrix observed in the crystal structure of *Ec*ThrRS. Figures were generated using the PyMOL Molecular Graphics System, Version 2.5 Schrödinger, LLC.

### Quantitative proteomics analysis

For proteomics analyses, three biological replicates of each yeast strain were prepared and analyzed. Log phase cells pre-cultured at 30°C were incubated at 37°C for 2 hours, and cell pellets from 20 ml cultures were fast frozen in liquid nitrogen and stored at −80°C. Protein extraction was prepared by beads beating in lysis buffer (100 mM HEPES pH 8.0, 8 M urea, 0.5% SDS, and 1× protease inhibitor). Supernatants were collected following centrifugation at 200 g for 20 min. Total proteins were quantified using BCA protein assay kit (Thermo Fisher Scientific). Tandem Mass Tag (TMT)-based multiplexed quantitative proteomics analysis was performed at The Thermo Fisher Center for Multiplexed Proteomics at Harvard University and analyzed on an Orbitrap Lumos mass spectrometer. MS2 spectra were searched using the COMET algorithm against a Yeast Uniprot composite database containing its reversed complement and known contaminants. For proteome, peptide spectral matches were filtered to a 1% false discovery rate (FDR) using the target-decoy strategy combined with linear discriminant analysis. The proteins were filtered to a <1% FDR and quantified only from peptides with a summed SN threshold of >180.

## RESULTS

### ThrRS editing-defective mutation results in serine misincorporation and heat sensitivity

**Figure 1.**
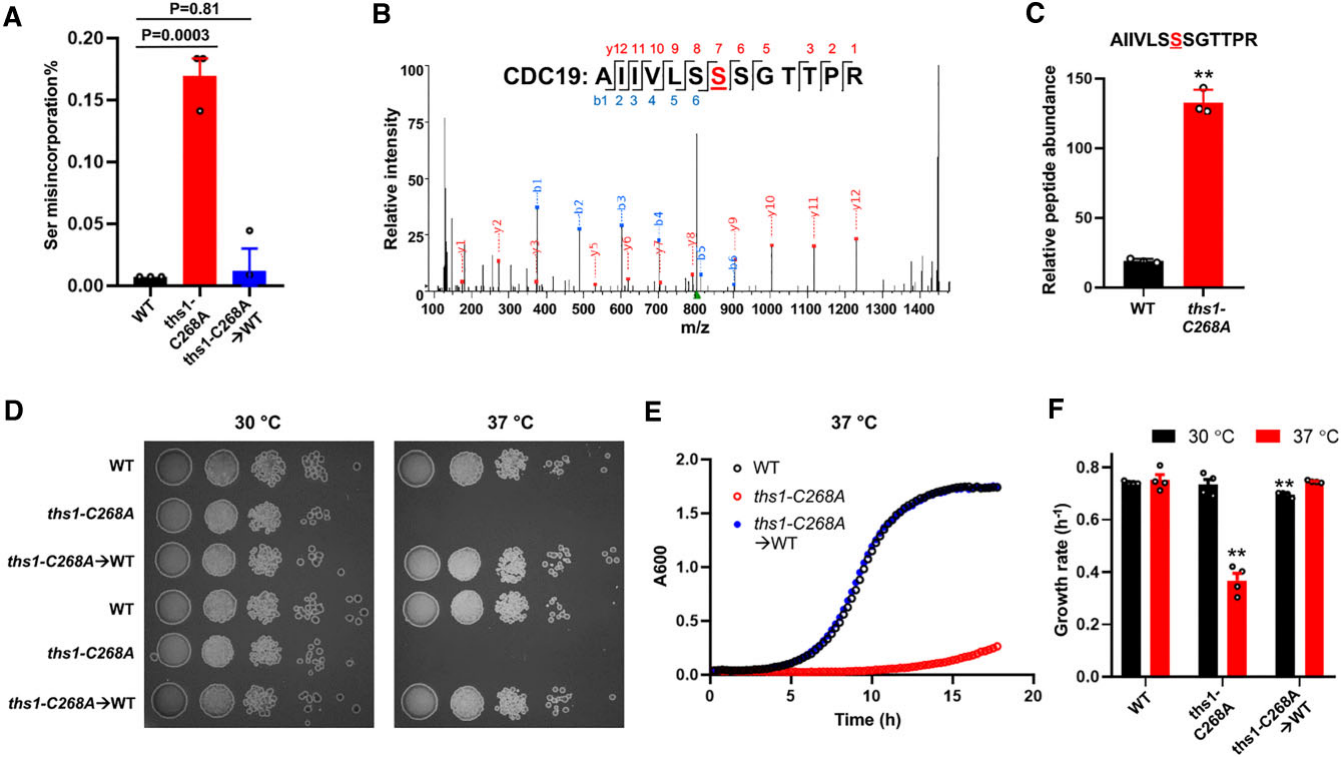
Editing-defective mutation in yeast ThrRS leads to heat sensitivity. (**A**) Ser misincorporation percentage in the WT, C719A and revertant yeast strains was determined with the β-lactamase reporter. Cells were grown in SD-Ura at 30°C. The percentage shows the activity ratio of the S68T β-lactamase variant over the WT β-lactamase. Misincorporation of Ser at Thr codons restores active β-lactamase for the S68T variant. (**B**) Tandem mass spectrometry of a peptide containing Ser misincorporation (highlighted in red) at a Thr codon. (**C**) Relative abundance of the peptide in (B). In TMT-based multiplexed quantitative proteomics analyses, proteins from different samples are labeled with different isobaric tags and mixed for tandem MS. The peptide shown here (from WT and *ths1-C268A* samples) was detected simultaneously as a single precursor ion peak, and the relative abundance was determined by the signals of the isobaric tags. (**D**) Yeast cells were grown in YPD to log phase and spotted on YPD agar plates with 10-fold dilutions. The plates were incubated for 2 days before imaging. The images shown here are representatives of at least three biological replicates. (**E**) Representative growth curves of yeast strains in YPD liquid medium out of at least three biological replicates. (**F**) Quantitation of growth rates in YPD. Statistical analysis compares the mutants with the WT at the same temperature. Error bars represent one standard deviation (SD) from the mean in at least three biological replicates. The *P* values are determined using the unpaired *t*-test. ** *P* < 0.01.

To understand the physiological role of ThrRS editing, we used a Clustered Regularly Interspaced Short Palindromic Repeats (CRISPR)-Cas9 system (40) to introduce a *C268A* mutation in the yeast *THS1* gene. Cys268 is conserved among bacterial and eukaryotic ThrRSs (Figure S1), and has been shown to be essential for editing in *E. coli* ThrRS (24,39). Mutating the equivalent residue (C182) in *E. coli* ThrRS causes the accumulation of Ser-tRNA^Thr^ and misincorporation of Ser at Thr codons (39,49). Using a β-lactamase reporter (43), we show that Ser misincorporation increases over 15-fold in the *ths1-C268A* mutant compared to the wild type (WT, Figure 1A), indicating an editing defect. Further proteomics analysis also identified an increased level of peptides with Thr to Ser substitutions in the *ths1-C268A* mutant (Figures 1B and C). Reverting the genomic *ths1-C268A* mutation with CRISPR-Cas9 restored Ser misincorporation to the WT level (Figure 1A).

We next tested the growth of the yeast variants in the rich medium yeast-extract-peptone-dextrose (YPD). The *ths1-C268A* mutant showed normal growth at 30°C but exhibited severe growth defects under heat stress at 37°C both on agar plates and in liquid media (Figures 1D–F). Reverting the *ths1-C268A* mutation fully restored growth at 37°C, ruling out any potential effect of non-specific mutations during genome editing. Previous work suggested that activation of the ISR is at least partially responsible for the peripheral neuropathy caused by GlyRS mutations (15,16), and mutations in glutamyl-prolyl-tRNA synthetase in diabetic patients also activate ISR (50). In yeast, ISR depends on Gcn2 to phosphorylate eIF2α (51,52). We found that abolishing ISR by deleting *GCN2* in the *ths1-C268A* mutant did not restore growth at 37°C (Figure S2), suggesting that ISR does not account for the heat sensitivity caused by the *ths1-C268A* mutation.

### Experimental evolution of suppressor mutants from *ths1-C268A* under heat stress

**Figure 2.**
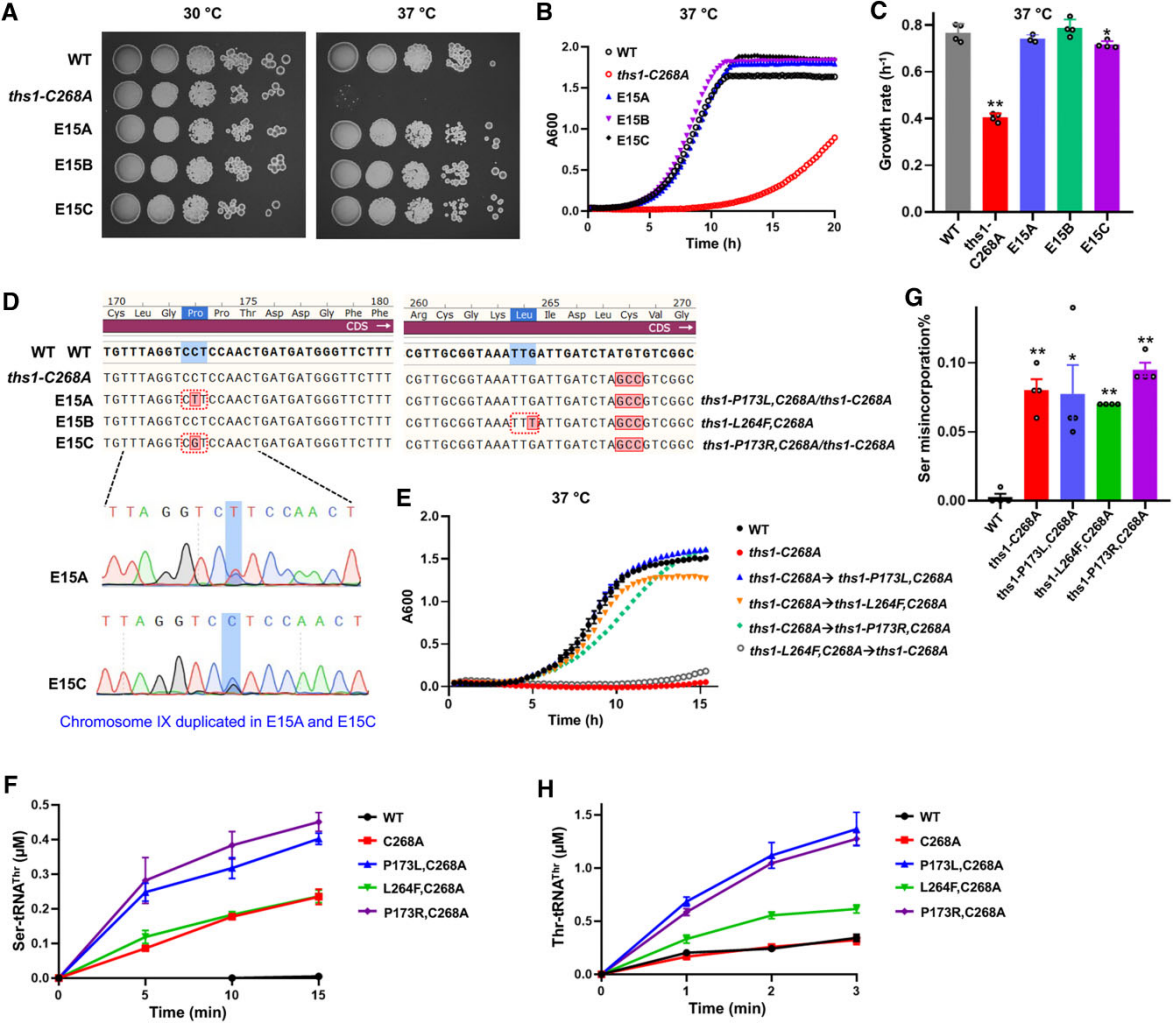
Intragenic mutations in ThrRS rescue heat sensitivity caused by the *ths1-C268A* mutation. (**A–C**) The growth of yeast variants was tested as in Figure 1. E15A-C are independently evolved strains from the *ths1-C268A* mutant after 15 cycles. The figures shown here are representatives of at least three biological replicates. (**D**) Sanger sequencing results of the *THS1* gene in the evolved strains. E15A and E15C strains carry two *THS1* alleles due to duplication of Chromosome IX. (**E**) Growth curves of yeast strains in YPD with means and standard deviations of at least three biological replicates. The mutations identified in the evolved strains are introduced chromosomally to the *ths1-C268A* strain using CRISPR-Cas9. (**F**) Ser misacylation by purified ThrRS (3 μM) *in vitro* with means and standard deviations of triplicates. Efficient editing in the WT prevents the accumulation of Ser-tRNA^Thr^ through hydrolysis. (**G**) Ser misincorporation percentage was determined with the β-lactamase reporter as in Figure 1A. (**H**) Thr aminoacylation by ThrRS variants (0.3 μM) with means and standard deviations of triplicates. Mutations in the evolved strains increase the aminoacylation efficiency. Error bars represent one SD from the mean. The *P* values are determined using the unpaired *t*-test. ** *P* < 0.01.

To understand how the ThrRS editing-defective mutation causes heat sensitivity, we performed three independent evolution experiments of the *ths1-C268A* strain at 37°C (Figure S3A). After 10 rounds (∼66 generations) of evolution, cells from two replicates had partially enhanced growth at 37°C (Figure S3B). Whole-genome sequencing results indicated that they contain a duplication of chromosome IX, which harbors the *ths1-C268A* allele (Figure S3C), but identified no other mutations that could explain the enhanced growth. This indicates that duplicating the partially functional *ths1-C268A* allele improved growth. To test whether duplication of *ths1-C268A* affects heat resistance, we expressed ThrRS variants from a low-copy plasmid in the *ths1-C268A* strain. An extra copy of *ths1-C268A* indeed partially rescued growth at 37°C (Figures S3D). Another replicate of experimental evolution (E10B) exhibited fully restored growth at 37°C after 10 rounds. Whole-genome sequencing shows that E10B contains a *ths1-L264F* mutation in addition to the *ths1-C268A* mutation (Figures 2D and S3C). Engineering this double mutant *ths1-L264F,C268A* from *the ths1-C268A* strain using CRISPR-Cas9 revealed that the additional L264F mutation was responsible for the restored growth (Figure 2E). After 15 rounds (∼100 generations), all three replicates (E15A, E15B and E15C) had evolved to restore growth at 37°C (Figures 2B-C and S3B). Whole-genome sequencing shows that E15B retained the *ths1-L264F* mutation as expected and maintained a single copy of chromosome IX. The E15A and E15C strains still contain the duplicated chromosome IX, but also each acquired an additional mutation at the P173 position of one copy of *THS1* (*ths1-P173L* and *ths1-P173R*, respectively, Figures 2D and S3C). To confirm that these additional *ths1* mutations restored heat resistance, we introduced them either in the genome of the *ths1-C268A* strain (Figure 2E) or as an extra *THS1* allele on a plasmid (Figure S3D). Our results demonstrate that secondary mutations of the *THS1* allele are responsible for fully rescuing the heat sensitivity caused by the *ths1-C268A* mutation.

### Suppressor mutations do not restore editing but enhance aminoacylation

The suppressor mutations are located in the editing domain of ThrRS, prompting us to test whether these mutations restore the editing activity. We expressed and purified His_6_-tagged yeast ThrRS variants from *Escherichia coli* and performed Ser misacylation experiments *in vitro*. The WT ThrRS did not accumulate mischarged Ser-tRNA^Thr^ due to efficient editing (Figure 2F). The C268A mutant yielded mischarged Ser-tRNA^Thr^ over time, supporting that C268 is essential for ThrRS editing. None of the suppressor mutations lowered mischarged Ser-tRNA^Thr^ formation (Figure 2F) or promotes deacylation of Ser-tRNA^Thr^ (Figure S4), suggesting that these secondary mutations did not restore the editing activity. In line with this, the suppressor strains show similar levels of Ser misincorporation as the *ths1-C268A* strain (Figure 2G). We further tested the Thr aminoacylation activity by ThrRS variants. Interestingly, we found that all three suppressor mutants exhibited increased levels of correctly charged Thr-tRNA^Thr^ at 37°C (Figure 2H).

### *ths1-C268A* mutation destabilizes ThrRS at 37°C

**Figure 3.**
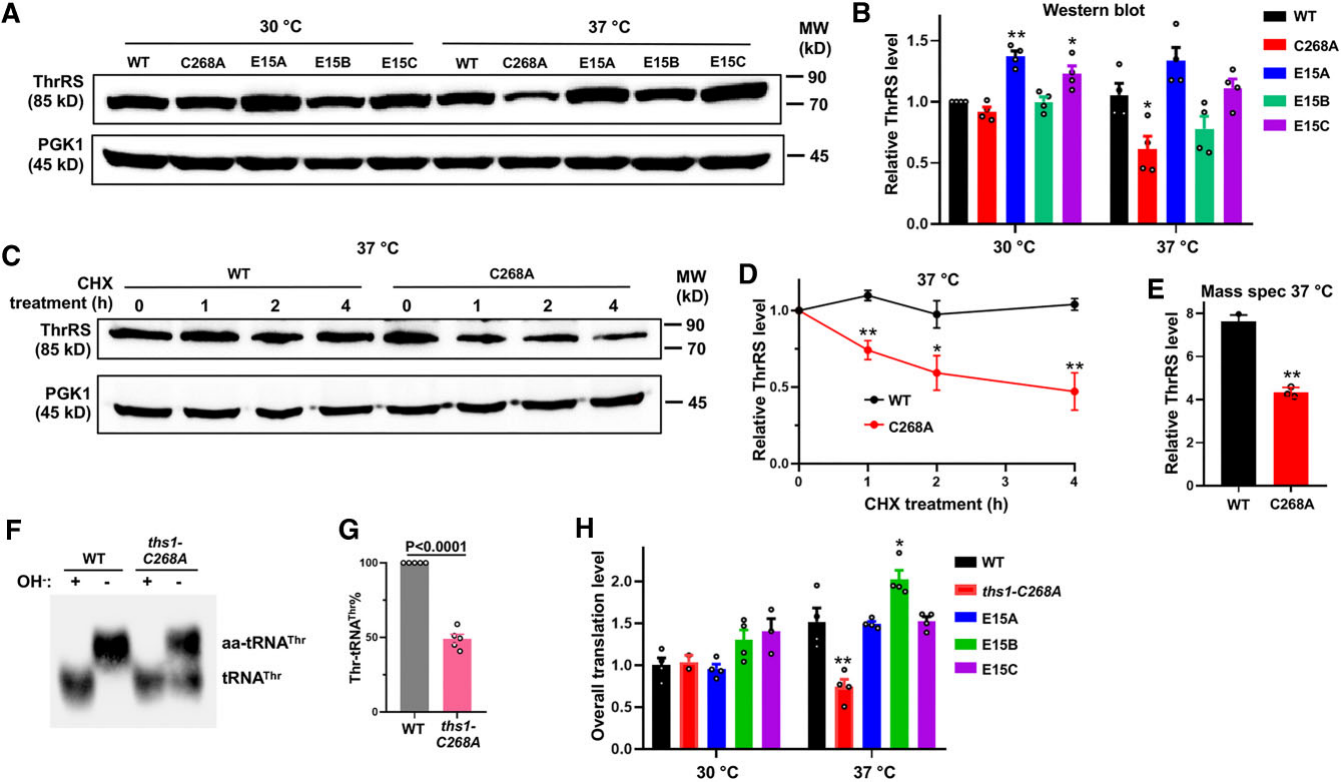
C268A destabilizes ThrRS and decreases overall protein synthesis at 37°C. (**A, B**) Western blot against FLAG-tagged ThrRS. Yeast cells were grown in YPD at 30°C to log phase and then incubated at 37°C for 2 hours before preparation of total proteins. The relative ThrRS level was normalized with the WT from Western blot experiments. Statistical analysis compares the mutants with the WT at the same temperature. (**C, D**) Degradation of ThrRS following the addition of 500 ng/μl of CHX to inhibit protein synthesis. The *ths1-C268A* mutation caused rapid degradation of ThrRS. (**E**) Relative ThrRS protein level revealed by TMT-based multiplexed quantitative proteomics. The Y-axis indicates the ThrRS percentage from each sample relative to total ThrRS in the protein mixture of all samples (also see Table S1). (**F, G**) Acidic northern blot against tRNA^Thr^. WT and *ths1-C268A* cells were grown at 30°C to mid-log phase and then incubated at 37°C for 2 hours. Alkaline (OH^−^) treatment of the total RNA causes deacylation. The *ths1-C268A* mutant shows a lower level of aminoacylation. (**H**) Overall protein synthesis level determined by the incorporation of a Met analog L-homopropargylglycine (HPG) in the proteome. Error bars represent one SD from the mean in at least three biological replicates. The *P* values are determined using the unpaired *t*-test. ** *P* < 0.01. The figures shown in (A, C, F) are representatives of at least three biological replicates.

ThrRS editing-site mutations found in human patients have been shown to lower the ThrRS protein level in patient-derived cell lines (30), leading us to test the ThrRS protein level of our yeast variants *in vivo*. We added a FLAG tag to the C-terminus of *THS1* and confirmed that the tag did not affect growth, and that the FLAG-tagged *ths1-C268A* strain still exhibited severe growth inhibition at 37°C (Figure S5). Western blot results revealed that the *ths1-C268A* mutation indeed decreased the ThrRS protein level by approximately 40% after 2 h of incubation at 37°C (Figure 3A). The evolved strains E15A and E15C (*ths1-P173L,C268A/ths1-C268A* and *ths1-P173R,C268A/ths1-C268A*) had a similar ThrRS protein level as the WT strain at 37°C. Whereas the ThrRS protein level is not fully restored, the increased ThrRS aminoacylation activity may compensate for the production of Thr-tRNA^Thr^ (Figure 2H). Next, we performed a time-course degradation assay following inhibition of protein synthesis with cycloheximide (CHX) and found that the ThrRS C268A protein was degraded faster than the WT at 37°C *in vivo*. Consistent with the Western blot result, quantitative proteomics analysis also shows that the *ths1-C268A* mutation decreases the ThrRS protein level (Figure 3E). Lowering ThrRS protein is expected to decrease aminoacylation and protein synthesis. To test this, we extracted total RNAs from the WT and *ths1-C268A* cells treated at 37°C for 2 h under acidic conditions to prevent aa-tRNA^Thr^ hydrolysis and performed acidic northern blot. As expected, the *ths1-C268A* cells had a significantly lower percentage of aa-tRNA^Thr^ level (Figure 3F and G). Additionally, the *ths1-C268A* strain exhibited a decrease in the overall protein synthesis compared to the WT (Figure 3H). These data indicate that the *ths1-C268A* mutation destabilizes the ThrRS protein, which is compensated by duplication of the *ths1-C268A* allele and secondary mutations in *THS1* in the evolved strains.

### ThrRS editing deficiency is not sufficient to cause heat sensitivity

**Figure 4.**
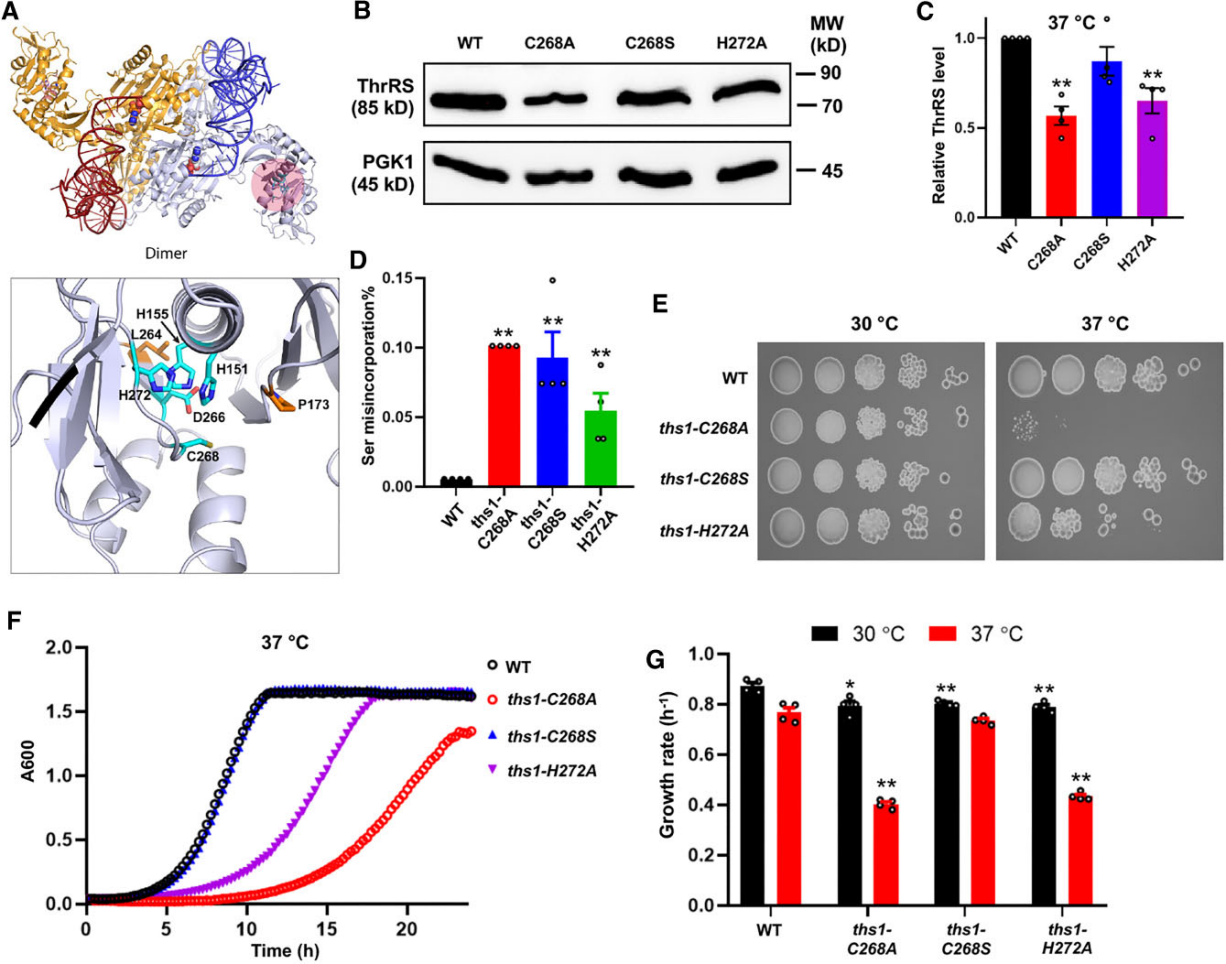
Effects of ThrRS editing-defective mutations on heat resistance. (**A**) Homology model of *S. cerevisiae* cytoplasmic ThrRS with bound tRNA and adenosine monophosphate (AMP). Ribbon diagram of the 3D model for *Sc*ThrRS using the crystal structure of *E. coli* ThrRS (PDB: 1QF6)(46) as the template. The two *Sc*ThrRS monomers in the two-fold symmetric dimer are shown in gold and lavender, with the bound tRNA in red and blue, respectively. Each *Sc*ThrRS monomer is bound to one molecule of AMP that is shown as a CPK model. The inset shows an enlarged view of Cys268 together with His151, His155, Asp266, and His272. The aforementioned residues are conserved from bacteria to humans. (**B, C**) ThrRS protein levels at 37°C revealed by Western blot as in Figure 3A. The relative ThrRS level was normalized with the WT. (**D**) Ser misincorporation levels determined with the β-lactamase reporter as in Figure 1A. (**E–G**) Growth of yeast variants as described in Figure 1. Statistical analysis compares the mutants with the WT at the same temperature. Error bars represent one SD from the mean in at least three biological replicates. The *P* values are determined using the unpaired *t*-test. ** *P* < 0.01. The figures shown in (B, E) are representatives of at least three biological replicates.

To dissect the contribution of aminoacylation and editing defects to heat sensitivity, we constructed additional editing-site mutants using CRISPR-Cas9. The 3D structure of yeast cytoplasmic ThrRS remains to be determined. To facilitate our mutant design, we generated a homology model of the yeast ThrRS *in silico* using the crystal structure of the *E. coli* ThrRS [PDB: 1QF6] (46) as a structural template. In our model, the side chain of C268 forms electrostatic interactions with neighboring charged residues that position a helix-hairpin-helix (Figure 4A). Disruption of the electrostatic network may destabilize the folding of the editing site. We predicted that the electrostatic interactions are impaired by the C268A change but maintained by the C268S mutation. In addition, His272 is also involved in the electrostatic interactions, and the H272A mutation may destabilize ThrRS. We found that as predicted, the more conservative C268S mutation did not significantly decrease the ThrRS protein level at 37°C but still increased Ser misincorporation (Figures 4B–D), indicating that the C268S change caused an editing defect as the C268A mutation. The H272A mutation both decreased the ThrRS protein level and increased Ser misincorporation, but to a lesser extent as compared to the C268A mutation. Intriguingly, the *ths1-C268S* mutant strain exhibited no growth defect at 30 or 37°C (Figures 4E–G). At 37°C, the *ths1-H272A* strain exhibited a growth defect compared to the WT but grew better than the *ths1-C268A* strain. These results suggest that ThrRS editing deficiency alone does not lead to growth defects under heat stress.

### Combined aminoacylation and editing defects cause heat sensitivity

**Figure 5.**
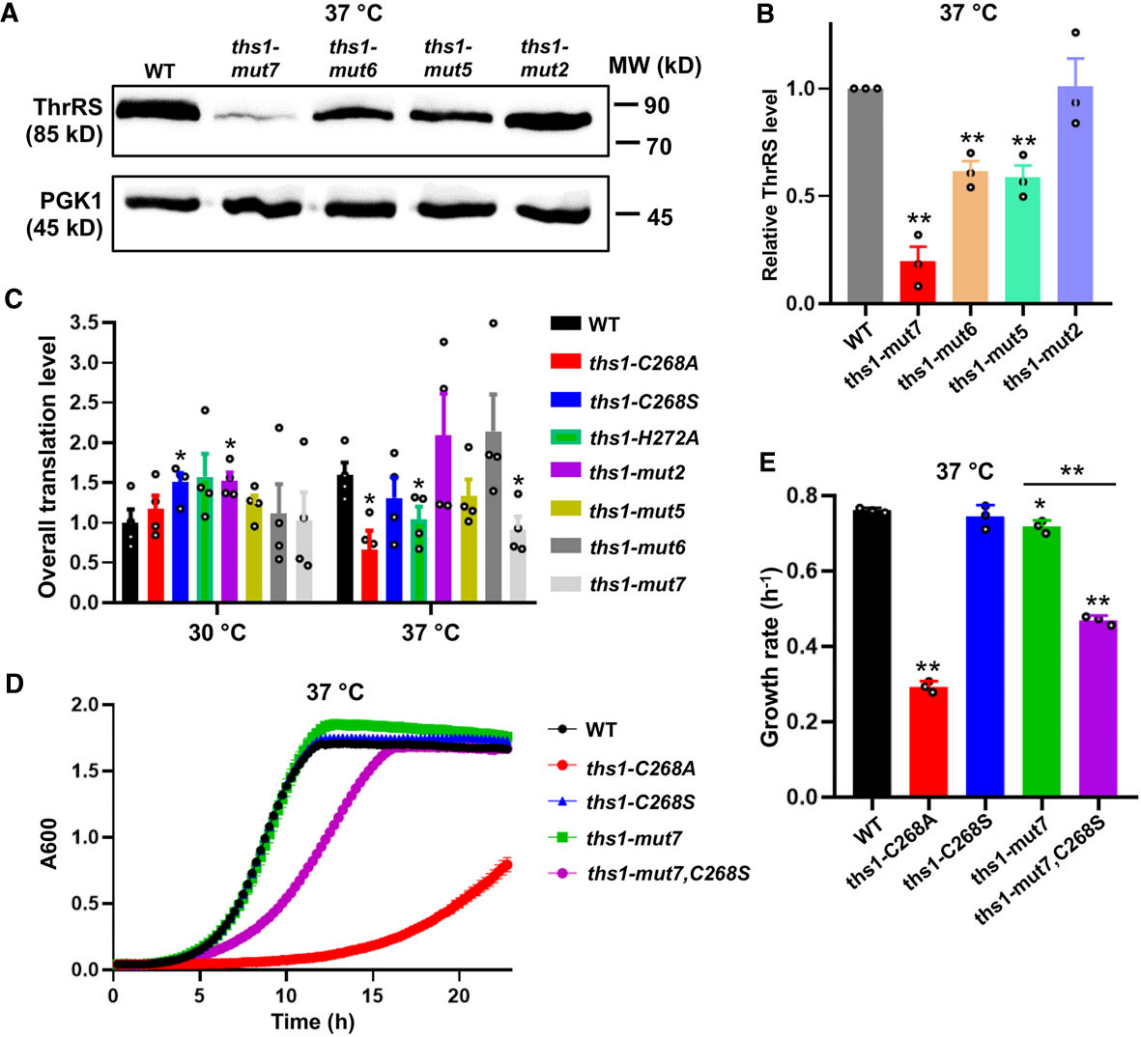
Aminoacylation and editing defects of ThrRS synergistically cause heat sensitivity. (**A, B**) Western blot against FLAG-ThrRS. Mut 5–7 show decreased ThrRS protein levels. The relative ThrRS level was normalized with the WT. The figure shown in (A) is representative of three biological replicates. (**C**) Protein synthesis level determined with HPG incorporation. Statistical analysis compares the mutants with the WT at the same temperature. (**D, E**) Growth of yeast variants. A combination of C268S and Mut 7 changes lead to a growth defect at 37°C. Error bars represent one SD from the mean in at least three biological replicates. The *P* values are determined using the unpaired *t*-test. ** *P* < 0.01.

We have shown that the *ths1-C268A* mutation decreases the ThrRS protein level at 37°C. To determine how varying ThrRS level affects heat sensitivity, we used CRISPR-Cas9 to randomize the nucleotide sequence 5′ to the translation start site of *THS1*. Western blot results revealed that several mutants exhibited various levels of ThrRS (Figures 5A and B). For instance, *ths1-mut7* had a lower level of ThrRS than the WT, and its overall protein synthesis level is similar to the *ths1-C268A* strain (Figures 5A–C). However, the growth of *ths1-mut7* was unaffected at 37°C (Figures 5D and E), suggesting that lowering the ThrRS protein level alone does not lead to heat sensitivity. We next combined the editing-defective *ths1-C268S* mutation with the reduced expression of *ths1-mut7*. The double mutant (*ths1-mut7,C268S*), which expressed a decreased ThrRS level as the *ths1-mut7* mutant (Figure S6), exhibited slower growth than the WT or *ths1-mut7* and *ths1-C268S* single mutant strains (Figures 5D, E, and S7). Collectively, our results support that combined aminoacylation and editing defects synergistically impair growth under heat stress.

### ThrRS C268A mutation dampens CAT tailing during ribosome-associated quality control

The *ths1-C268A* mutation decreased the overall protein synthesis level (Figure 5C). To determine the abundance of individual proteins, we performed multiplexed quantitative proteomics of the WT, *ths1-C268A*, *ths1-C268S*, and *ths1-mut7* strains treated at 37°C for 2 h (Figure S8). Using multiplexed isobaric labeling, a total of over 4400 proteins were identified for each tested strain (Table S1). We did not observe significant changes in stress-activated chaperones that assist protein refolding between the WT and *ths1-C268A* mutant (Figure S8C), indicating that the capacity to deal with misfolded proteins is not enhanced in the *ths1-C268A* strain.

**Figure 6.**
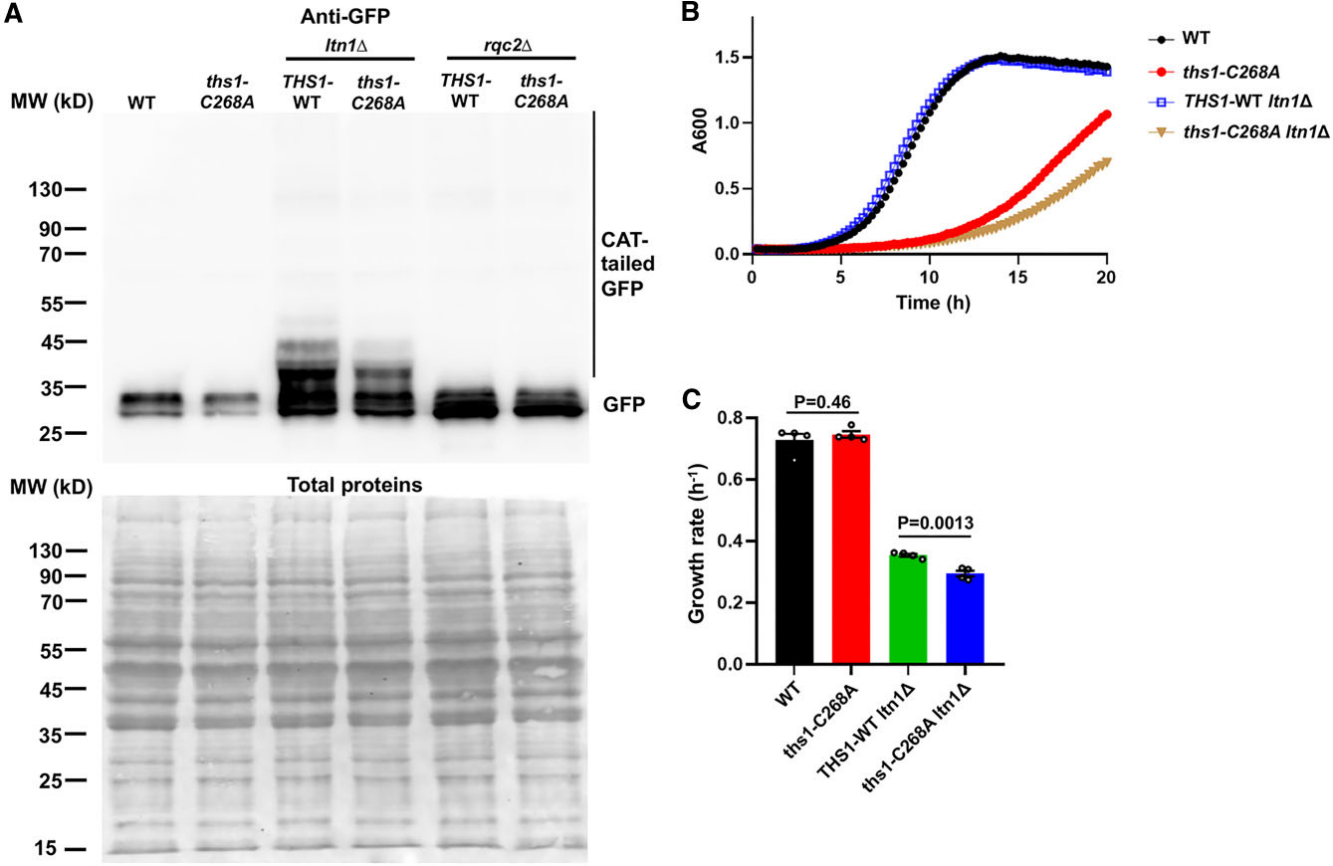
ThrRS C268A mutation dampens CAT tailing during RQC. (**A**) Detection of CAT tails with the pTDH3-GFR-R12-RFP reporter. Cells were grown at 30°C to mid-log phase and then incubated at 37°C for 5 hours prior to Western blot using an anti-GFP antibody. Ribosome stalling at the R12 motif leads to CAT tailing of GFP. Deleting *LTN1* prevents ubiquitination and proteasome degradation of CAT-tailed proteins, and deleting *RQC2* abolishes CAT tailing. The *ths1-C268A* mutant shows a lower level of CAT tailing, consistent with a decreased supply of Thr-tRNA^Thr^. Total proteins are detected with Ponceau staining of the same membrane following Western blot. Representative images of three biological replicates are shown. (**B, C**) Deleting *LTN1* further decreases growth in the *ths1-C268A* mutant at 37°C. Error bars represent one SD from the mean in at least three biological replicates. The *P* values are determined using the unpaired t-test.

In addition to translation, Thr-tRNA^Thr^ is also used as a substrate in RQC process for adding CAT tails to stalled polypeptides (31,36). CAT tailing has been shown to drive the degradation of stalled polypeptides both on the ribosome in an Ltn1-dependent manner and off the ribosome, thereby protecting cells against proteotoxic stress (36,37). We have shown that the *ths1-C268A* mutation decreases the aminoacylation level of tRNA^Thr^ (Figure 3F and G). We next examined CAT tail formation using an established GFP reporter (34) with ribosome stalling poly-arginine sequences. In the absence of Ltn1, which ubiquitinates stalled polypeptides for proteasome degradation, CAT tailing was indeed impaired in the *ths1-C268A* mutant (Figure 6C). Deleting *LTN1* further decreased the growth of the *ths1-C268A* strain at 37°C but had no effect on the growth of the WT strain (Figures 6B and C). Collectively, these results suggest that the *ths1-C268A* mutation leads to aminoacylation deficiency and impaired CAT tailing during RQC.

### ThrRS aminoacylation and editing defects predispose cells to aminoglycoside toxicity

Heat stress causes global protein misfolding and proteotoxicity (34,53). To determine how ThrRS aminoacylation and editing defects impact cellular defense against other proteotoxic stresses, we tested the growth rates of yeast variants in the presence of an aminoglycoside G418, which slows ribosomal translation and increases translational errors (54,55). We show that all three editing-defective mutants (*ths1-C268A*, *ths1-C268S* and *ths1-H272A*), as well as *ths1-mut7*, are all more sensitive to G418 treatment (Figure S9), further supporting our notion that robust aminoacylation and editing are critical to defending against proteotoxicity.

## DISCUSSION

Aminoacylation is a fundamental process in all living organisms and provides the ribosome with aa-tRNA building blocks for protein synthesis. Decades of work has provided insights into the structural basis and kinetic mechanisms of aminoacylation and editing, as well as noncanonical functions of aaRSs beyond protein synthesis (5,9,56,57). On the other hand, we are only beginning to understand the physiological impact of aminoacylation and editing, which is critical not only to uncover the regulatory mechanisms of gene expression and stress responses but also to elucidate the molecular basis of human diseases caused by mutations in aaRSs. Recent advances in exome and whole-genome sequencing have revealed a rapidly growing number of aaRS mutations that lead to human diseases (11,12). Compared to dominant aaRS mutations that cause peripheral neuropathy, recessive mutations in cytoplasmic aaRSs lead to disorders in the central nervous system. We previously reported that compound heterozygous mutations in glutaminyl-tRNA synthetase (GlnRS) cause progressive microcephaly and brain atrophy (19). Since this discovery, pathogenic recessive mutations have been found in 17 (out of 20) other cytoplasmic aaRSs in patients with neurological disorders (11,12). Most of these mutant aaRSs are reported to exhibit a lower aminoacylation efficiency, decreased protein stability or both, leading to the speculation that their disease-causing mechanisms may be due to loss of aaRS function. Exactly how aminoacylation defects lead to cellular toxicity is unclear. In several cases, protein misfolding has been implicated to worsen the disease onset caused by recessive aaRS mutations (21). For instance, GlnRS mutations found in microcephaly patients cause increased ubiquitination and aggregation of the GlnRS protein (19), and a pathogenic mutation in AlaRS has been shown to cause an editing defect (21). Neurons are particularly sensitive to proteotoxic stress (58). It is therefore tempting to speculate that aminoacylation deficiency may cause severe damage to cells in combination with proteotoxic stress. In this study, we demonstrate that combined aminoacylation and editing defects lead to severe proteotoxicity.

ThrRS and AlaRS share homologous editing domains to hydrolyze misacylated Ser-tRNAs (22,24). Editing-defective AlaRS mutations have been shown to cause neurodegeneration in mice and heat sensitivity in *E. coli* and yeast (29,43,59). Interestingly, deleting one AlaRS allele further enhances proteotoxicity in editing-defective mice (28), suggesting that as in our ThrRS study, combining editing defects with reduced protein levels enhances phenotypic consequences. In humans, the K276E and L227P mutations in the ThrRS editing site are associated with developmental disorders and intellectual disability (30). These mutations appear to destabilize ThrRS in cells, and the equivalent mutations in yeast cause lethality (30). These studies are consistent with our findings that aminoacylation and editing defects synergistically sensitize cells to proteotoxic stress.

**Figure 7.**
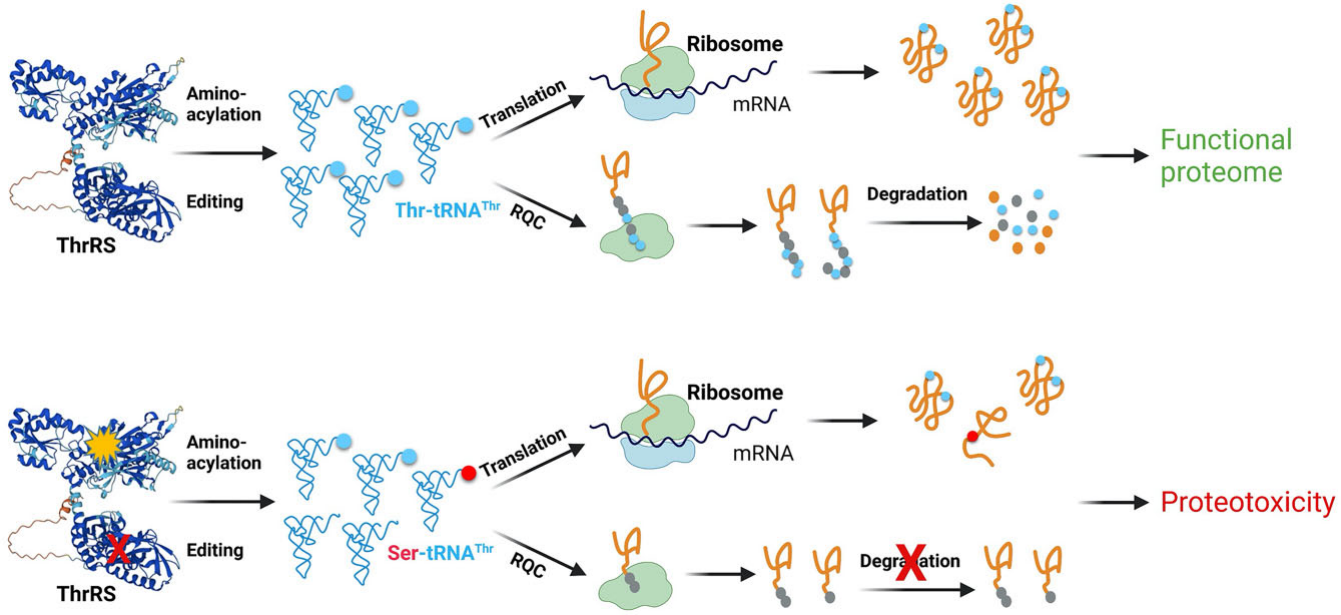
Model of proteotoxicity resulting from concerted aminoacylation and editing defects of ThrRS. Robust aminoacylation and editing of ThrRS lead to an efficiently and accurately translated proteome, which is functional and protected against proteotoxic stresses, such as heat. An aminoacylation defect in ThrRS lowers the supply of Thr-tRNA^Thr^, which leads to both decreased protein synthesis and impaired RQC. An editing defect further increases Ser misincorporation and protein misfolding, thereby contributing to the overall proteotoxicity. Created with BioRender.com.

Efficient aminoacylation by ThrRS and AlaRS is required to provide Thr-tRNA^Thr^ and Ala-tRNA^Ala^ substrates to RQC, which results in CAT tailing of stalled polypeptides. A recent study suggests that Rqc2 binding to aa-tRNAs is a rate-limiting step in CAT tailing, which is slower than canonical translation (60). RQC and CAT tailing are critical for cellular defense against proteotoxicity (31,34–37). Mutations in NEMF (the mammalian homolog of Rqc2), which catalyzes the mRNA-independent addition of Ala and Thr to the stalled polypeptides, result in neurological defects and a shortened life span in mice (38). Such mutations are also associated with juvenile neuromuscular diseases in humans (38). CAT tails have been proposed to extend the peptide from the ribosomal exit tunnel and expose Lys residues for ubiquitination by Ltn1 (37), and also directly serve as a degron to target polypeptides for degradation (36). In addition, some CATylated polypeptides were shown to form aggregates, possibly by sequestering toxic premature polypeptides (35). Here, we show that the *ths1-C268A* mutation decreases the aminoacylation level of tRNA^Thr^ and impairs CAT tailing (Figure 6). This may further increase the proteotoxicity in addition to the protein misfolding stress caused by ThrRS editing deficiency and heat (Figure 7).

## Supplementary Material

gkad778_Supplemental_Files

## Data Availability

The mass spectrometry proteomics data have been deposited with the ProteomeXchange Consortium via the PRIDE (61) partner repository under the dataset identifier PXD042145. Genome sequencing reads are deposited in Sequence Read Archive (SRA) under accession number PRJNA972286. Any additional information required to reanalyze the data reported in this paper is available from the lead contact upon request.
