# Supplementary material for: Coordination between aminoacylation and editing to protect against proteotoxicity
Source: Nucleic Acids Res. 2023 Sep 23;51(19):10606–18. doi: 10.1093/nar/gkad778 (PMC10602869; doi:10.1093/nar/gkad778)
Supplement: gkad778_Supplemental_Files [file gkad778_supplemental_files.zip › SI_Zhang et al.pdf]

Coordination between aminoacylation and editing to protect against proteotoxicity

Hong Zhang[1], Parker Murphy[1], Jason Yu[1], Sukyeong Lee[2,3], Francis T.F. Tsai[2,3,4,5], Ambro van Hoof[6], Jiqiang Ling[1*]

[1]Department of Cell Biology and Molecular Genetics, The University of Maryland, College Park, MD 20742, USA
[2]Department of Biochemistry and Molecular Biology, Baylor College of Medicine, Houston, TX 77030, USA
[3]Advanced Technology Core for Macromolecular X-ray Crystallography, Baylor College of Medicine, Houston, TX 77030, USA
[4]Department of Molecular and Cellular Biology, Baylor College of Medicine, Houston, TX 77030, USA
[5]Department of Molecular Virology and Microbiology, Baylor College of Medicine, Houston, TX 77030, USA
[6]Department of Microbiology and Molecular Genetics, The University of Texas Health Science Center at Houston, Houston, TX 77030, USA
*Correspondence should be addressed to:
Jiqiang Ling: +1 (301) 405-1035; Email: jling12@umd.edu

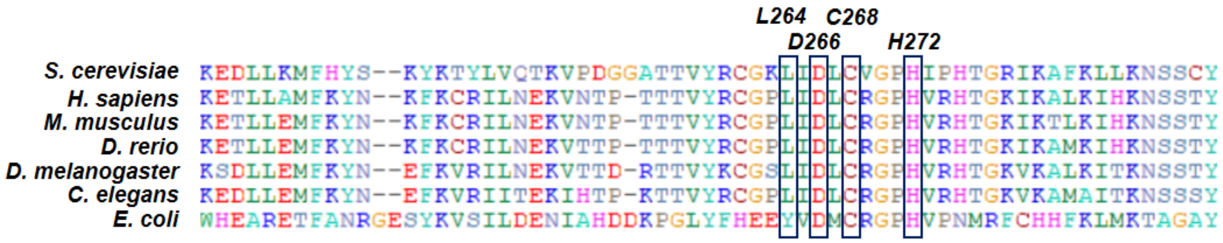

Supplemental Figure S1. Amino acid sequence alignment of the ThrRS editing site. The numbering is based on *S. cerevisiae* cytoplasmic ThrRS.

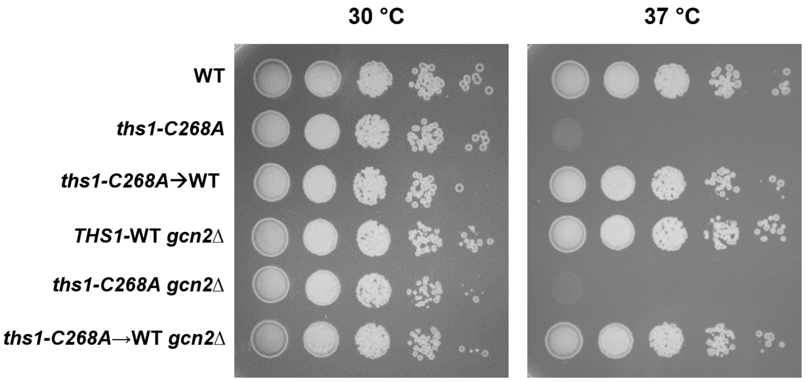

Supplemental Figure S2. Heat sensitivity of the ThrRS C268A mutant is independent of GCN2. Growth of yeast variants on YPD agar plates. Representative images of at least three biological repeats are shown. Deleting *GCN2* does not rescue the heat sensitivity caused by the *ths1-C268A* mutation.

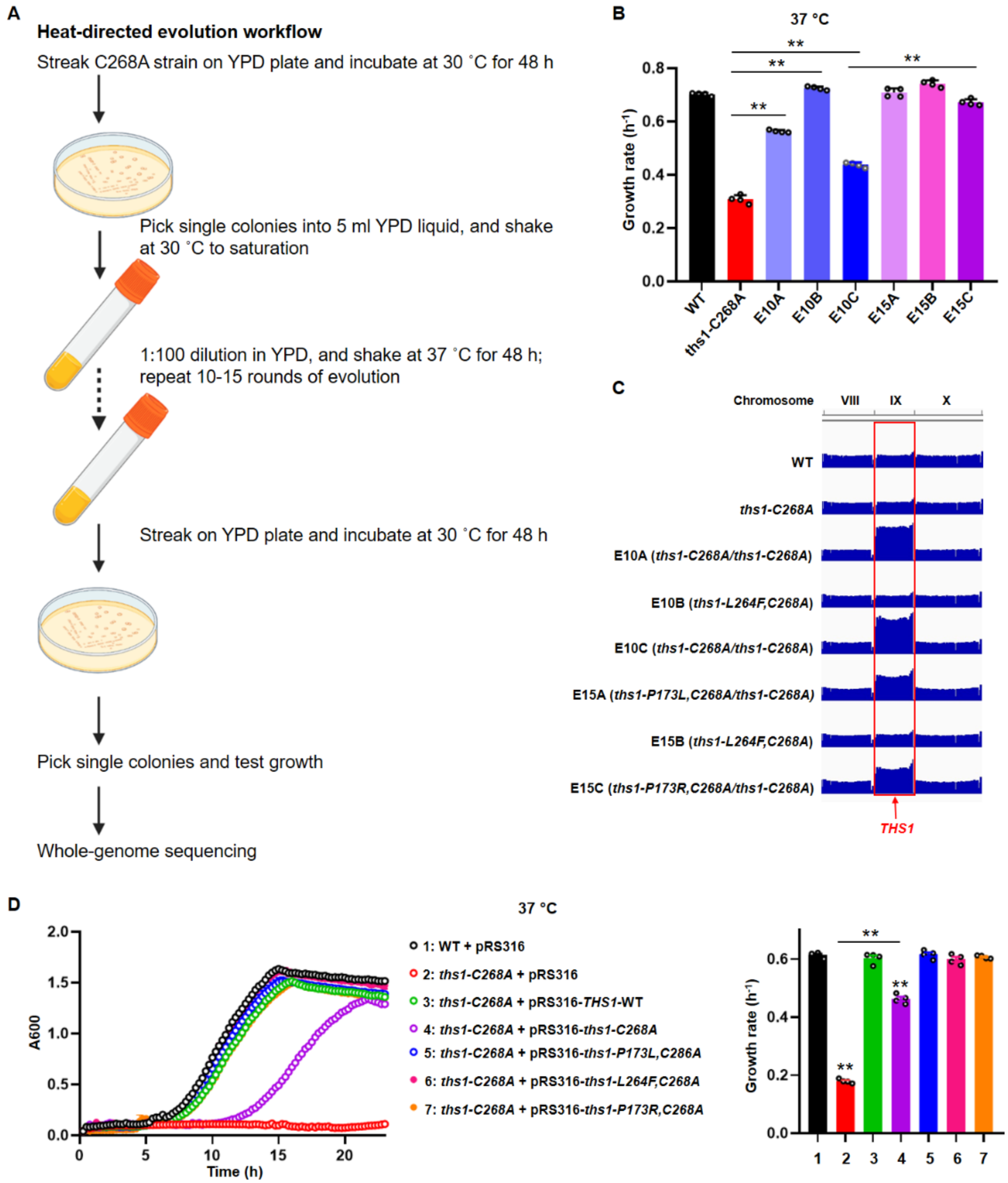

Supplemental Figure S3. Experimental evolution of suppressor mutants from ThrRS C268A. (A) Workflow of heat-directed evolution. (B) Growth rates of evolved strains in YPD at 37 °C. (C) Whole-genome sequencing shows duplication of chromosome IV in E10A, E10C, E15A, and E15C strains. (D) Growth of WT and *ths1-C268A* strains carrying a single-copy plasmid in SD - Ura media. Expressing an extra copy of the *ths1-C268A* allele partially restores heat resistance. Error bars represent one SD from the mean. The P values are determined using the unpaired t-test.

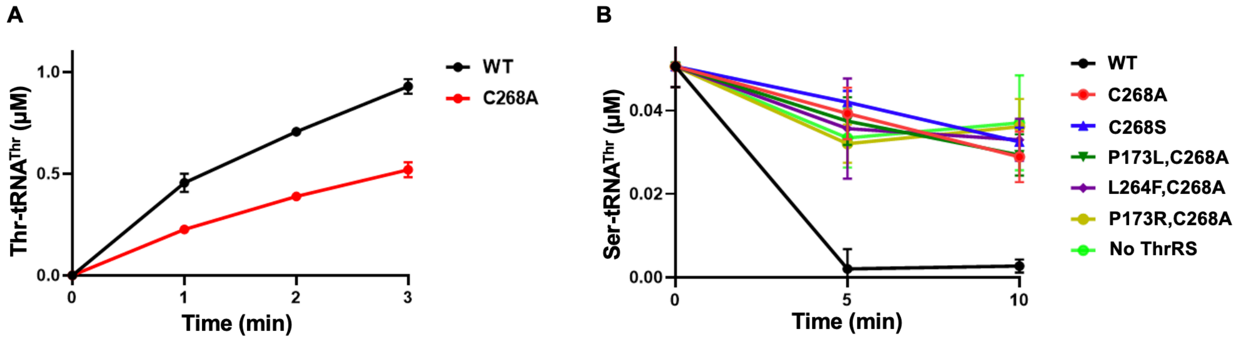

Supplemental Figure S4. Aminoacylation and deacylation by ThrRS variants. (A) Thr aminoacylation by ThrRS variants (0.3 μM) with means and standard deviations of triplicates. ThrRS was pre-incubated at 37 °C for 2 hours before the reaction starts. (B) Deacylation of pre-synthesized Ser-tRNA^{Thr} (50 nM) by ThrRS variants (1 μM) at 37 °C with means and standard deviations of triplicates.

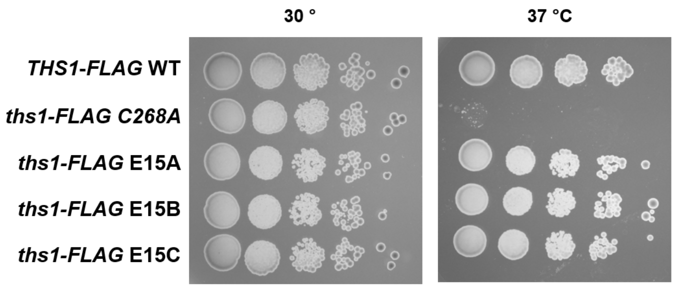

Supplemental Figure S5. Growth of yeast strains with FLAG-tagged ThrRS. FLAG tagging of ThrRS does not affect growth. Representative images of three biological replicates are shown.

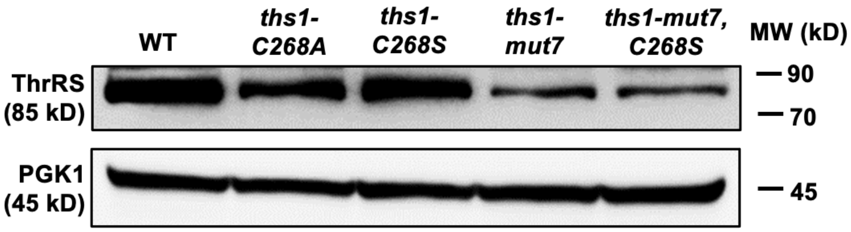

Supplemental Figure S6. Western blot against FLAG-tagged ThrRS. Yeast cells were grown in YPD at 30 °C to log phase and then incubated at 37 °C for 2 hours before preparation of total proteins. The figure is representative of three biological replicates.

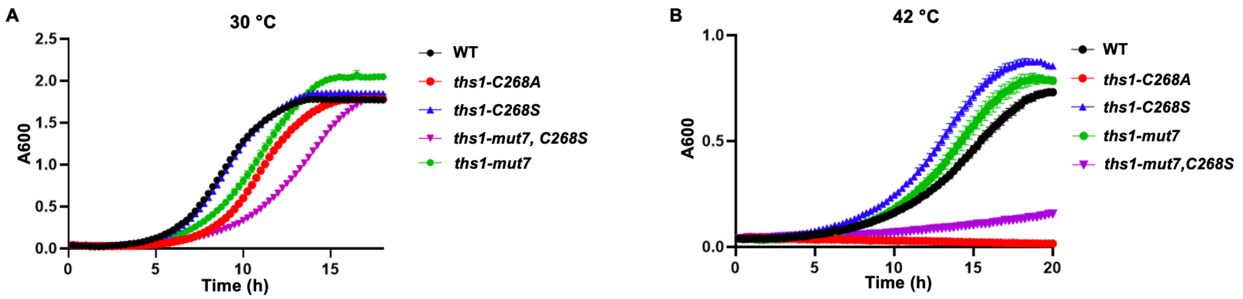

Supplemental Figure S7. Growth curves of yeast strains in YPD with means and standard deviations of at three biological replicates at (A) 30 °C and (B) 42 °C. The *ths1-mut7,C268S* double mutant grows worse than either *ths1-mut7* or *ths1-C268S* at both temperatures, suggesting synergistic toxicity of ThrRS aminoacylation and editing defects.

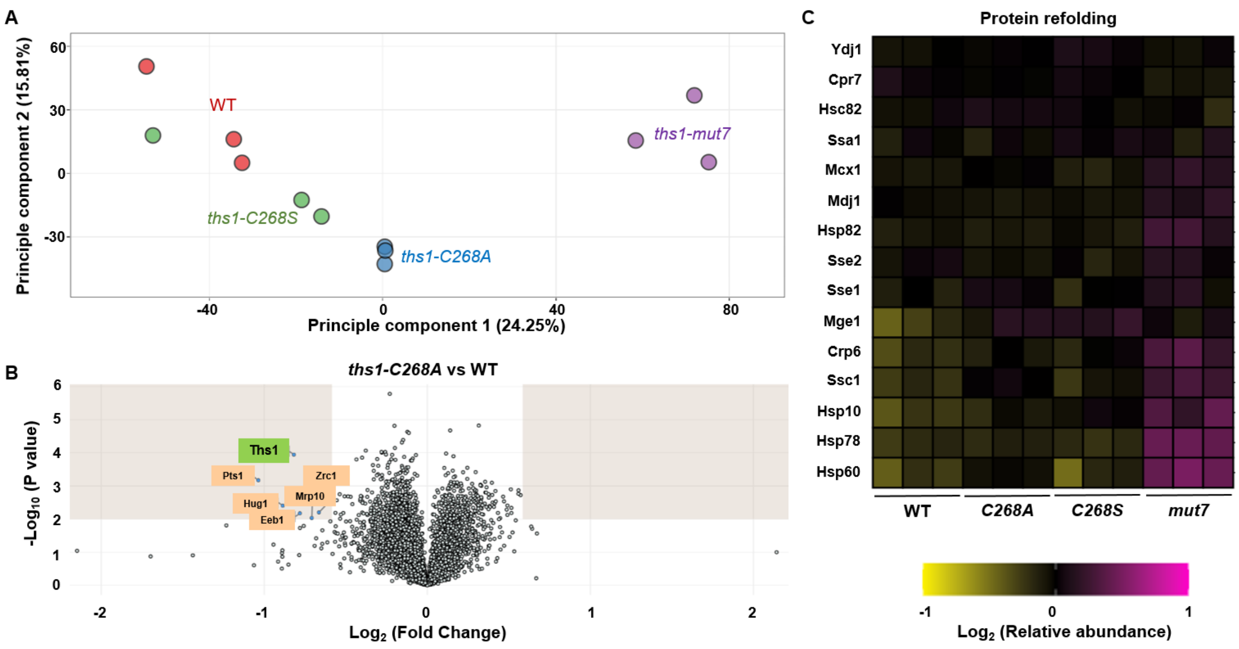

Supplemental Figure S8. Quantitative proteomics of yeast strains using multiplexed isobaric labeling. Yeast cells were grown in YPD at 30 °C to log phase and incubated at 37 °C for 2 hours before total proteins were prepared. Three biological replicates were tested for each strain. (A) Principal component analyses. (B) Volcano plot comparing identified proteins in the WT and *ths1-C268A* strains. The cut-off for significant changes is 1.5-fold with a P value of <0.01. (C) Heat map of relative levels of chaperones involved in protein refolding.

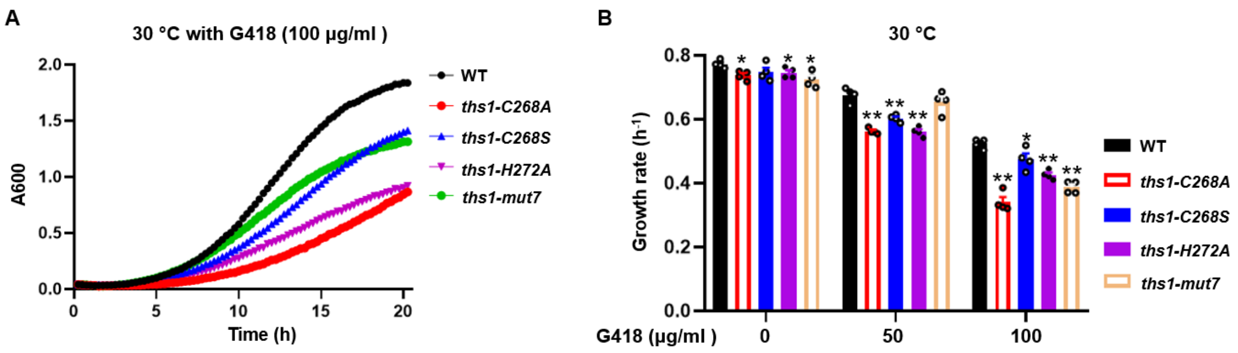

Supplemental Figure S9. ThrRS aminoacylation and editing-defective mutants are more sensitive to aminoglycoside. Yeast cells were grown in YPD at 30 °C in the presence and absence of G418. (A) Representative growth curves out of at least three biological replicates. (B) Calculated growth rates. Statistical analysis compares the mutants with the WT at the same concentration of G418. Error bars represent one SD from the mean. The P values are determined using the unpaired t-test. ** P < 0.01.

Supplementary Table S1. Quantitative proteomics data of yeast variants.

Supplementary Table S2. Strains and plasmids used in this study.

**Strain or Plasmid**	**Relevant properties**	**Sources**
**Strain**		
E. coli DH5α	Used for plasmids amplification	Ling lab
Rosetta(DE3)pLysS	Used for protein expression	Ling lab
WT	Haploid *Saccharomyces cerevisiae* strain BY4741	Ling lab
ths1-C268A	ThrRS mutant derived from BY4741	This study
ths1-C268A →WT	Revertant of *ths1-C268A*	This study
THS1-WT *gcn2Δ*	*GCN2* was replaced by *LEU2*	This study
ths1-C268A gcn2Δ	*GCN2* was replaced by *LEU2*	This study
ths1-C268A →WT *gcn2Δ*	*GCN2* was replaced by *LEU2*	This study
THS1-WT *ltn1Δ*	*LTN1* was replaced by *HIS3*	This study
ths1-C268A ltn1Δ	*LTN1* was replaced by *HIS3*	This study
THS1-WT rqc2Δ	*RQC2* was replaced by *HIS3*	This study
ths1-C268A rqc2Δ	*RQC2* was replaced by *HIS3*	This study
ths1-C268S	ThrRS mutant derived from BY4741	This study
ths1-H272A	ThrRS mutant derived from BY4741	This study
E10A	Evolved strain from *ths1-C268A* after 10 rounds	This study
E10B	Evolved strain from *ths1-C268A* after 10 rounds	This study
E10C	Evolved strain from *ths1-C268A* after 10 rounds	This study
E15A	Evolved strain from *ths1-C268A* after 15 rounds	This study
E15B	Evolved strain from *ths1-C268A* after 15 rounds	This study
E15C	Evolved strain from *ths1-C268A* after 15 rounds	This study
THS1-FLAG WT	*THS1* FLAG-tagged strain	This study
ths1-FLAG C268A	*THS1* FLAG-tagged strain	This study
ths1-FLAG C268S	*THS1* FLAG-tagged strain	This study
ths1-FLAG H272A	*THS1* FLAG-tagged strain	This study
ths1-FLAG E15A	*THS1* FLAG-tagged strain	This study

ths1-FLAG E15B	*THS1* FLAG-tagged strain	This study
ths1-FLAG E15C	*THS1* FLAG-tagged strain	This study
ths1-mut2	Mutant yeast strain with altered 5’ UTR of *THS1*	This study
ths1-mut5	Mutant yeast strain with altered 5’ UTR of *THS1*	This study
ths1-mut6	Mutant yeast strain with altered 5’ UTR of *THS1*	This study
ths1-mut7	Mutant yeast strain with altered 5’ UTR of *THS1*	This study
ths1-mut7/ths1-C268S	Double mutant	This study
**Plasmid**		
pET28a	Protein expression vector	Ling lab
pRS316	Expression vector for yeast, *URA3*	Ling lab
pTDH3-GFP-R12-RFP	RQC reporter, *URA3*	Brandman lab
pCas9	Cas9 expression, *LEU2*	Addgene
pJD1212	Cherry-Venus expression, *URA3*	Dinman lab
pJD-bla	β-lactamase expression, *URA3*	Ling lab
pJD-bla-S68T	β-lactamase S68T expression, *URA3*	This study
pRS316-bla	β-lactamase expression, *URA3*	This study
pRS316-bla- S68T	β-lactamase S68T expression, *URA3*	This study
psgRNA_C268	Expression of sgRNA targeting *THS1* C268, *HIS3*	This study
psgRNA_C268A	Expression of sgRNA targeting *tsh1-C268A*, *HIS3*	This study
psgRNA_THS1-IRES	Expression of sgRNA targeting *THS1* 5’ UTR region, *HIS3*	This study
pET28a-thrRS-WT	ThrRS WT expression and purification from *E. coli*	This study
pET28a-thrRS-C268A	ThrRS C268A expression and purification from *E. coli*	This study
pET28a-thrRS-E15A	ThrRS C268A,P173L expression and purification from *E. coli*	This study
pET28a-thrRS-E15B	ThrRS C268A,L264F expression and purification from *E. coli*	This study
pET28a-thrRS-E15C	ThrRS C268A,P173R expression and purification from *E. coli*	This study
pRS316-*THS1*-WT	ThrRS expression in yeast, *URA3*	This study
pRS316-*ths1-C268A*	ThrRS expression in yeast, *URA3*	This study
pRS316-*ths1-P173L,C268A*	ThrRS expression in yeast, *URA3*	This study
pRS316- *ths1-L264F,C268A*	ThrRS expression in yeast, *URA3*	This study

pRS316- *ths1-P173R,C268A*	ThrRS expression in yeast, *URA3*	This study

Supplemental Table S3. Oligos used in this study.

Oligo name	Sequences (5'-3')	Description
GCN2-KO-Leu F	GATTTTTTTTTTCAATAATTTTCCGTTCCCCTTAACACATACTATGTATAACCGCAGTTAACTGTGGGAATA	Amplification of *LEU2* to obtain *gcn2*Δ
GCN2-KO-Leu R	TACTTTACCTTTAACTGATGCGTTATAGCGCCGCACAGATCTTTAAAGGCATCACGTTGAGCCATTAGTATC	
GCN2-KO VF	AATCTTGACGTGCAAGGGCC	To verify and select *gcn2*Δ strains
GCN2-KO VR	ACCTACCCCCTTTACAACGGT	
ltn1-KO UF	CCAACACGACAATCATGAGTA	Amplification of *HIS3* to obtain *gcn2*Δ
ltn1-KO UR	TAAGAACCAACTATTGCTTGAAC	
HIS3 F	TTCAAGCAATAGTTGGTTCTTAgagcttg gtgagcgctag	
HIS3 R	cagaatgacacgtatagaatgatg	
ltn1-KO DF	atcattctatacgtgtcattctgCGAGGTTGTATT GAATGATAAGG	
ltn1-KO DR	ACTGATAAAGTGTATGGCCC	
ltn1-KO VF	CATCATTTGCGTCATCTTC	To verify and select *ltn1*Δ
ltn1-KO VR	AGATGAACTCTGAGACAAAC	
Rqc2-KO His F	AAAGAAAGTTGCTACTTATTATCCGGTCTAAGAAGTCAGGCAGGCAAGAGATTAATAGCGgagcttggtgagcgctag	Amplification of *HIS3* to obtain *gcn2*Δ
Rqc2-KO His R	ATGAAAAAATAATGAAAAAATTATAATTGCTGTCTATTTTCTTTTCATCTCATATGATTTAcagaatgacacgtatagaatga	
Rqc2-KO VF	GGCTTTCCAGGATAGCTA	To verify and select *rqc2*Δ.
Rqc2-KO VR	CGAGGATCATTTAGTCCTTTA	
Bla S68T F	AAGAAAGATTCCCAATGATGACCACTTTCAAGGTCTTGTTGTG	To mutate the *bla* gene to S68T

Bla S68T R	CATCATTGGGAATCTTTCTTCTGGTCTGAAAGATTCC	
PsgRNA-C268 F	cagtgaaagataaatgatcAAATTGATTGATCTATGTGTgttttagagctagaaatagc	Amplification to obtain the psgRNA_C268
psgRNA R	gatcatttatctttcactgcggagaagtttcgaacgc	
thrRS C268 UF	GTGAATCTTGTGAGTGCCACC	Amplification of *THS1* C268 synonymous donor DNA
thrRS C268synonymous UR	ATGTGGGATATGAGGGCCGACGCACAGGTCTATTAACTTACCGCAACGGTAGACGGT	
thrRS C268 DF	CGGCCCTCATATCCCACATAC	
thrRS C268 DR	CCTCTCTTACGGTATTCGGTTC	
PsgRNA-C268synonymous F	cagtgaaagataaatgatcAAGTTAATAGACCTGTGCGTgttttagagctagaaatagc	Amplification to obtain the psgRNA_C268_synonymous
psgRNA R	gatcatttatctttcactgcggagaagtttcgaacgc	
thrRS C268 UF	GTGAATCTTGTGAGTGCCACC	Amplification of *ths1-C268A* donor DNA
thrRS C268 muta UR	ATGTGGGATATGAGGGCCGACggcTAGATCAATCAATTTAC	
thrRS C268 DF	CGGCCCTCATATCCCACATAC	
thrRS C268 DR	CCTCTCTTACGGTATTCGGTTC	
psgRNA-thrRS_C268A	cagtgaaagataaatgatcAAATTGATTGATCTAgccGTgtttagagctagaaatagc	Amplification to obtain the psgRNA_C268A
psgRNA R	gatcatttatctttcactgcggagaagtttcgaacgc	
thrRS C268 DF	CGGCCCTCATATCCCACATAC	Amplification of *ths1-C268S* donor DNA
thrRS C268S UR	ATGTGGGATATGAGGGCCGACAGACAGGTCTATTAACTTACCGCAACGGTAGACGGT	
ThrRS H272A UF	GTGAATCTTGTGAGTGCCACC	Amplification of *ths1-H272A* donor DNA
ThrRS H272A UR	GTATGTGGGATggcAGGGCCGACGCACAGGTCTATTAACTTACCGCAACGGTAGACGGT	
ThrRS H272A DF	TCGGCCCTgccATCCCACATACTGGGCGT	
ThrRS H272A DR	CCTCTCTTACGGTATTCGGTTC	
PsgRNA-thS1 IRES F	cagtgaaagataaatgatcAACAGAGCAAGAAAATAAAAgttttagagctagaaatagc	
psgRNA R	gatcatttatctttcactgcggagaagtttcgaacgc	
ThrRS IRES UF	GGACAAGCGCCTATGGAAGTC	
ThrRS IRES UR	AGCTGTTAATTTTTCGTTAACCTG	

ThrRS IRES DF1	TTAACGAAAAATTAACAGCTAATAGGGCTAGGAAGTATAATCATGATGAGTGCTAGTGAAGCAGG	Amplification of the psgRNA-*THS1* 5'UTR and the UTR mutation donor DNA
ThrRS IRES DF2	TTAACGAAAAATTAACAGCTAATAGGGCTAGGAAGTATAATAGGCATGAGTGCTAGTGAAGCAGG	
ThrRS IRES DF3	TTAACGAAAAATTAACAGCTAATAGGGCTAGGAAGTATAAGTTAGATGAGTGCTAGTGAAGCAGG	
ThrRS IRES DF4	TTAACGAAAAATTAACAGCTAATAGGGCTAGGAAGTATAATTTGGATGAGTGCTAGTGAAGCAGG	
ThrRS IRES DR	GCTTCGCCTTCAAATGGTCTAT	
Psg-thS1-TAA F	cagtgaaagataaatgatcAACTTGAAAGATGAAAAGAGgttttagagctagaaatagc	Amplification to obtain the psgRNA-*THS1* and FLAG donor DNA
psgRNA R	gatcatttatctttcactgcggagaagtttcgaacgc	
thsFlag UF	GTTGTGCCAGTTGGTGTCAAGT	
ths1Flag synonymous UR	cttgtcgtcatcgtctttgtagtcAGCTAAGACGTTGTCACCTCTTTTCTCGTCCTTTAAATTACGCAACTGTTTCAAAACTTC	
thsFlag DF	acaaagacgatgacgacaagTAATGAGATTTTATGTAGTTAAATTTTGAC	
thsFlag DR	CAAATATCAGCGTTTACTGCAGAG	
thrRS C268 muta VF	AGGTCTTTTGGCATTCGTCTG	To verify C268A/C268S/H272A mutation
thrRS C268 muta VR	TGACCTGGACAGTTCATTGGTT	
Seq1F	GGACAAGCGCCTATGGAAGTC	Sanger sequencing of the *THS1* gene in the evolved strains to confirm the genome sequencing results
Seq2F	AGGTCTTTTGGCATTCGTCTG	
Seq3F	GAATTGCCATGGAGAGTTGCAG	
Seq4R	ATCTCAGGATTTTCTGACGATGGT	
tRNAThr_AGT	Biotin-TTGAACCGATGATCTCCACA	For northern blot
pRS316THS1 F	actcactatagggcgaattggagctccCTACCTCAGATAACAATGTCGAC	Plasmid construction
pRS316THS1 R	tcgaggtcgacggtatcgataagcttATCTCAGGATTTTCTGACGATG	
pET28a-ScThrRS F	gcctggtgccgcgcggcagccatatgAGTGCTAGTGAAGCAGGTGTC	Plasmid construction
pET28a-ScThrRS R	cagtggtggtggtggtggtgctcgagAGCTAAGACGTTGTCACCTCTC	
pET28a-ScThrRS C268A-P173L F	GTGCCCATATTTGTTTAGGTCTTCCAACTGATGATGGGTTCTT	Plasmid construction

pET28a-ScThRrS C268A-P173L R	ACCTAAACAAATATGGGCACCTAGGTGGCACTCACAA	
pET28a-ScThrRS C268A-P173R F	GTGCCCATATTTGTTTAGGTCGTCCAACTGATGATGGGTTCTT	Plasmid construction
pET28a-ScThrRS C268A-P173R R	ACCTAAACAAATATGGGCACCTAGGTGGCACTCACAA	
pET28a-ScThrRS C268A-L264F F	CCGTCTACCGTTGCGGTAAATTTATTGATCTAGCCGTCGGCCC	Plasmid construction
pET28a-ScThrRS C268A-L264 R	TTTACCGCAACGGTAGACGGTAGTAGCACCTCCATCT	
